# VDAP-GUI: a user-friendly pipeline for variant discovery and annotation of raw next-generation sequencing data

**DOI:** 10.1007/s13205-016-0382-1

**Published:** 2016-02-15

**Authors:** Ramesh Menon, Namrata V. Patel, Amitbikram Mohapatra, Chaitanya G. Joshi

**Affiliations:** Department of Animal Biotechnology, College of Veterinary Sciences and Animal Husbandry, Anand Agricultural University, Anand, Gujarat 388 001 India

**Keywords:** High-throughput sequencing, Single nucleotide polymorphism, INDEL mutation, Software pipeline

## Abstract

Even though next-generation sequencing (NGS) has become an invaluable tool in molecular biology, several laboratories with NGS facilities lack trained Bioinformaticians for data analysis. Here, focusing on the variant detection application of NGS analysis, we have developed a fully automated pipeline, namely Variant Discovery and Annotation Tool-Graphical User Interface (VDAP-GUI), which detects and annotates single nucleotide polymorphisms and insertions/deletions from raw sequence reads. VDAP-GUI consolidates several proven methods in each step such as quality control, trimming, mapping, variant detection and annotation. It supports multiple NGS platforms and has four methodological choices for variant detection. Further, it can re-analyze existing data with alternate thresholds and generates easily interpretable reports in html and tab-delimited formats. Using VDAP-GUI, we have analyzed a publically available human whole-exome sequence dataset. VDAP-GUI is developed using Perl/Tk programming, and is available for free download and use at http://sourceforge.net/projects/vdapgui/.

**Electronic supplementary material:**

The online version of this article (doi:10.1007/s13205-016-0382-1) contains supplementary material, which is available to authorized users.

## Introduction

In the last decade the massively parallel next-generation sequencing (NGS) technology has revolutionized life science research, which pave the way for several landmark discoveries (Metzker 2010; Shendure and Ji 2008). For example, a single experiment can identify thousands of single nucleotide variants (SNVs) and small INDELs associated to a genetic disorder (Stitziel et al. 2011). With the introduction of affordable desktop NGS platforms several small and medium sized laboratories use this technology for whole genome/exome, transcriptome or metagenome applications (Gullapalli et al. 2012). Even though some NGS data analysis pipelines in the free domain are frequently updated to handle the huge datasets, more efficient and user-friendly analytical solution need to be evolved, especially in variant discovery applications. Present challenges in NGS data analysis includes computation power, storage, data sharing, expensive commercial softwares and requirement of skilled professionals (Sarovich and Price 2014). This apart, the fragmented nature of NGS softwares makes a comprehensive analysis out of reach for many life science researchers. Although few automated pipelines have been developed recently, many of them lack a user-friendly interface or are limited by functionality (Blanca et al. 2011; D’Antonio et al. 2013; Sarovich and Price 2014).

Here, we have developed VDAP-GUI, an open-source tool for variant detection in eukaryotic genome/exome data. VDAP-GUI wraps several well-validated open-source programs into a single-platform, thereby simplifying and standardizing the analysis workflow. The methods in VDAP-GUI include FastQC (www.bioinformatics.babraham.ac.uk/projects/fastqc/) for quality control, PRINSEQ (prinseq.sourceforge.net/) for trimming, Burrows-Wheeler Aligner (BWA) (Li and Durbin 2010) for reference mapping. For SNP/INDEL detection it offers three standard methods such as SAMtools (Li et al. 2009), VarScan (Koboldt et al. 2009) and FreeBayes (Garrison and Marth 2012). Further, the variant annotation is carried out using variant effect predictor—VEP tool (http://www.ensembl.org/info/docs/tools/vep), which supports annotation of 65 eukaryotes.

## Materials and methods

### Tools used in VDAP-GUI and development of pipeline

The methods in VDAP-GUI include FastQC—version 0.11.2 (www.bioinformatics.babraham.ac.uk/projects/fastqc/) for quality control and PRINSEQ—version 0.20.4 (prinseq.sourceforge.net/) for quality filter and trimming. For reference mapping, BWA-mem (version 0.7.5a) algorithm has been utilized (Li and Durbin 2010). The SNP/INDEL detection methods used in VDAP-GUI were: SAMtools (version 0.1.19), VarScan (version 2.3.7) and FreeBayes (version 0.9.10-3) (Garrison and Marth 2012; Koboldt et al. 2009; Li et al. 2009). Further, a custom approach namely, *MultiCom* is also introduced to detect SNPs detected by more than one method. The *VCF*-*tools intersect* method has been used to derive *MultiCom* results. For variant annotation, Ensembl VEP tool version 78 (http://www.ensembl.org/info/docs/tools/vep) was employed, which supports annotation of 65 eukaryotes.

Initially, the tools used in VDAP-GUI were compiled on Ubuntu Linux (version 14.04) and integrated into a pipeline using Perl language (version 5.20), followed by testing it in the command line version. Next, the code was subjected to necessary modification after addition of Tk module (version 804.032) for GUI mode. In the pipeline, some in-house Perl/bash scripts were used for the automation process.

## Results and discussion

### Overview of VDAP-GUI workflow

**Fig. 1:**
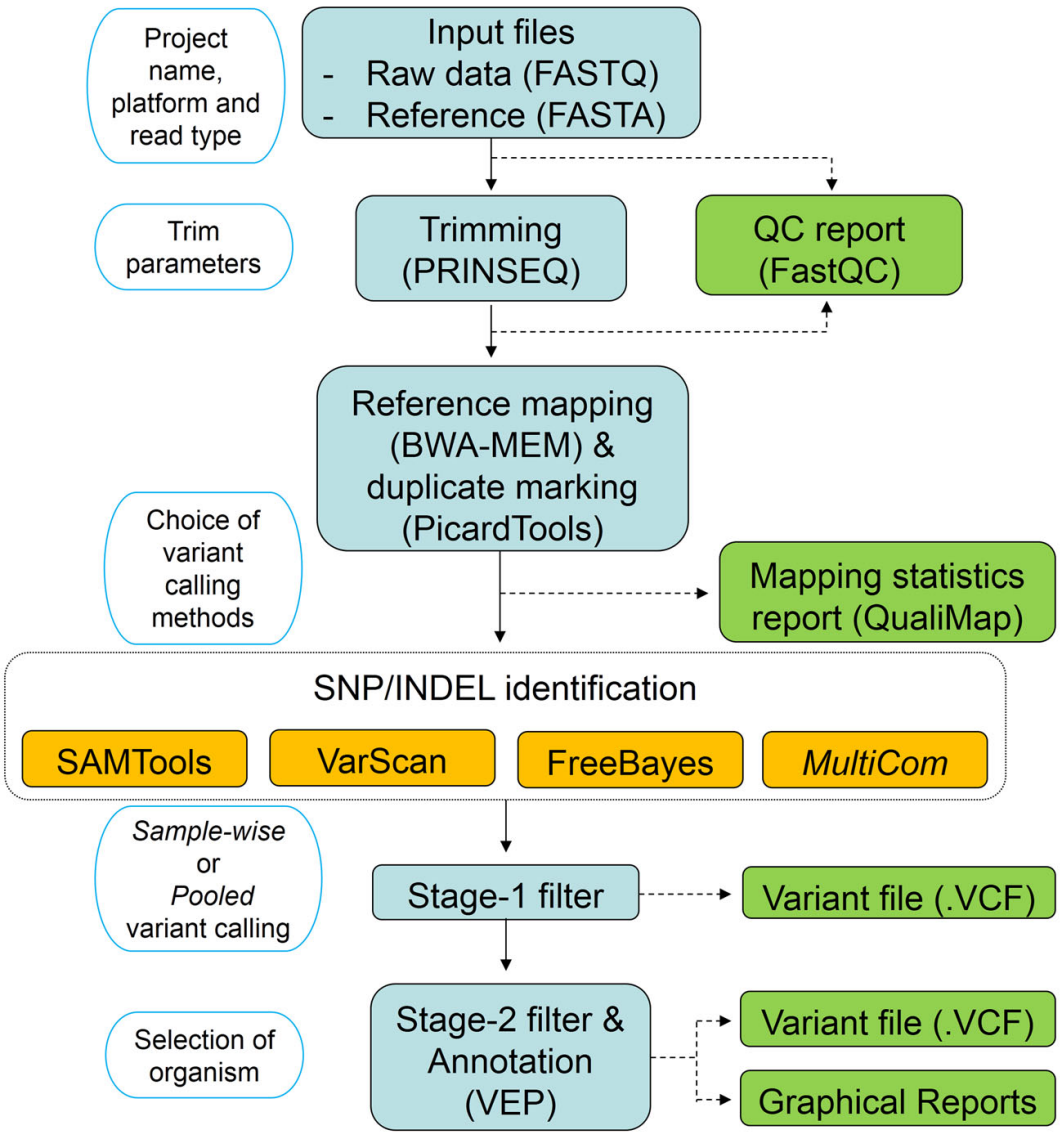
(*Workflow of VDAP*-*GUI*) the *left section* shows the user input to the software. The *middle section* indicates the processes and softwares used. In the variant calling process four options are possible *S* SAMtools, *V* VarScan, *F* FreeBayes and *M* Multicom. The *right section* shows the outputs given at each step by VDAP-GUI. The *arrows* indicate dependency between each step. The *upper panel* shows the analysis pipeline and the *lower panel* shows the variant annotation module

The VDAP-GUI tool wraps a collection of publicly available NGS analysis tools using Perl/Tk programming. The workflow of VDAP-GUI pipeline is depicted in Fig. 1, which consists of five steps: (1) *create project/import input files*, (2) *QC and Trimming*, (3) *reference mapping*, (4) *SNP/INDEL identification*, (5) *annotation*. Further, this menu-driven software is highly customizable at different steps of analysis.

### Creating project and input file import

**Fig. 2:**
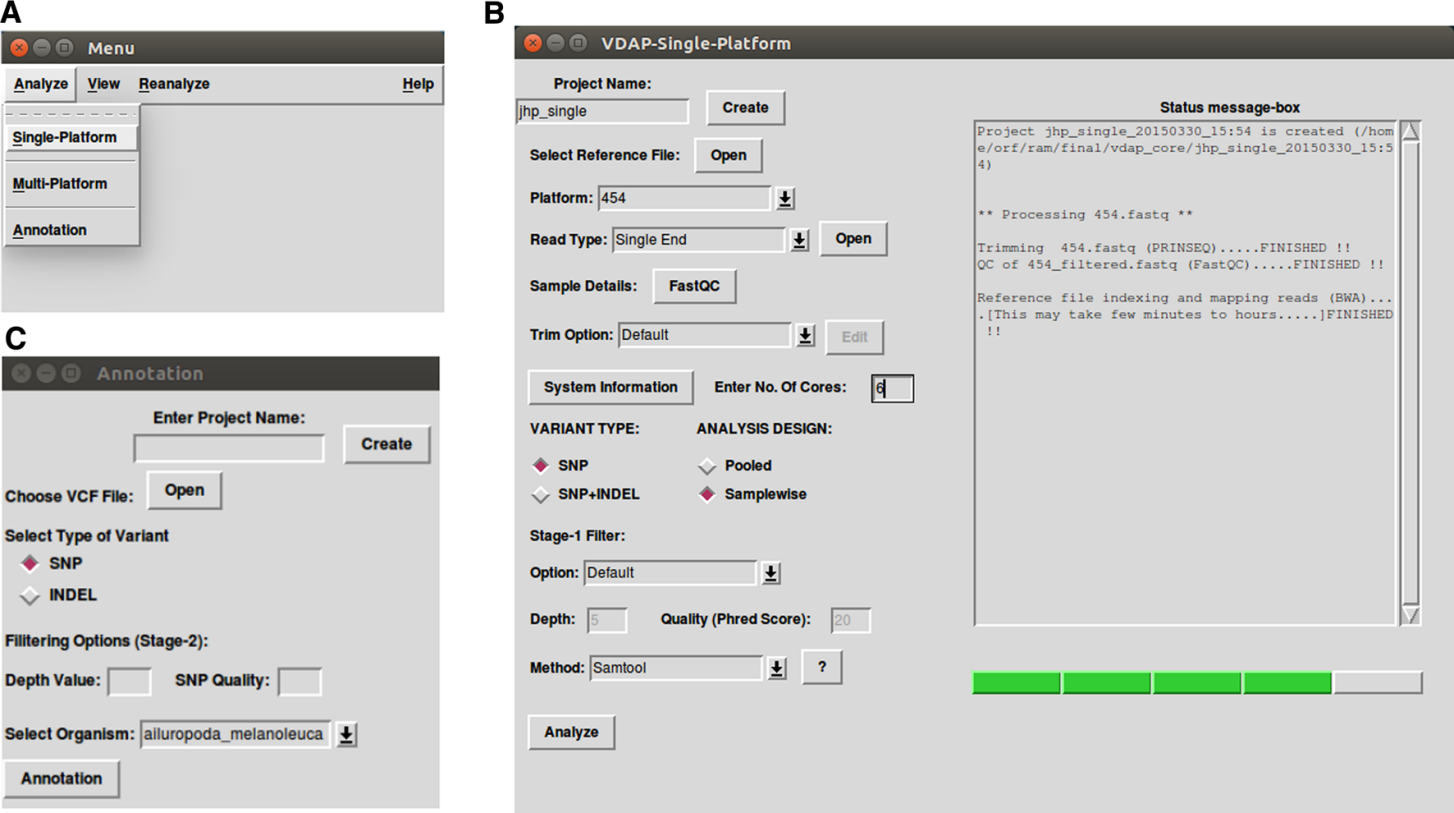
(*Screenshots of VDAP*-*GUI*) the *panel A* shows the main Menu of VDAP-GUI. The *Analyze* menu has items such as *Single*-*platforms*, *Multi*-*platform* and *Annotation.* The *View* menu has *Results* item. The Re-*analyze* menu has items such as *Single*-*platforms* and *Multi*-*platform.* The *Help* has *Manual*, *Credits*, and *About* items. The *panel B* shows the *Analyze* → *Single*-*platform* module, in which the *left* side has the input and parameter setting and *right* hand side has a status message-box, to display the real-time status of the analysis along with a progress *bar*. In the *Panel C*, *Annotation* module is given. User is prompted to provide the project name and choose the .vcf file along with the Stage 2 filter (optional). In addition, the user is provided with a drop-down list for the selection of organisms (currently, 65 eukaryotes are supported for annotation)

The analysis of raw sequence data can be evoked by selecting the *Analyze* item from the main menu (Fig. 2a). The pipeline needs to be initialized with a project creation, followed by selection of raw read dataset (FASTQ format) and a reference sequence file in FASTA format. In addition, appropriate platform name and read type (single or paired-end) need to be selected from the drop-down list (Fig. 2a).

### QC and trimming

In the QC analysis, the VDAP-GUI makes assessments about the quality of the reads, read length, GC content, k-mer analysis using FASTQC tool. Though optional, QC analysis of the raw reads is recommended to understand the overall quality of the dataset and estimate the parameters for filtering of low quality reads. However, QC will be performed in background of the filtered dataset. In both the cases, detailed reports will be stored in the project folder (Fig. 2b). The filtering of raw reads is performed using PRINSEQ tool, in which the user can choose between *default* and *custom* option. The *default* trim options are specific for each NGS platform (Fig. 2b). However, the filtering of raw reads can be skipped by choosing the appropriate parameters.

### Reference mapping and duplicate marking

The reliable reads are mapped to the reference genome in this step using bwa-mem algorithm. Initially, the workflow checks whether the reference sequence is indexed or not. If not, indexing will be performed using tabix tool. After reference mapping, the alignment file will be subjected to duplicate marking using Picard tools. The resultant SAM file will be converted to BAM format and in the project folder. In general, reference mapping and duplicate marking are the most time consuming step in VDAP-GUI.

### SNP/INDEL identification

The choice of the variant calling algorithm is one important factor that determines the reliability of the SNP/INDEL. VDAP-GUI provides four choices for SNP discovery, among which three are widely used algorithms and one is a strategy. The algorithms are SAMtools, VarScan and Freebayes. The strategy is named as *MultiCom*, which performs the variant discovery using all the three algorithms and selects those which are identified by at least two algorithms. At present, *MultiCom* feature is not implemented for INDEL discovery. VDAP-GUI reports variants based on the base depth and quality parameters given by the user, named as *Stage*-*1 filter*. The resultant file (.vcf) will be saved in the project folder.

### Annotation

Variant annotation is the final step in the VDAP-GUI. This step is implemented as an independent module, as this feature can also be utilized for annotation of variants obtained by tools other than VDAP-GUI (Fig. 2c). In this step, the user may opt for a second level of filtering (namely *Stage*-*2 filter*), to select the most reliable variants for annotation. The annotation is performed using VEP tool, which supports 65 eukaryotes. Apart from the report generated by VEP tool, VDAP-GUI provides detailed reports on variant prediction in html and csv formats.

### Additional features

VDAP-GUI offers the *re*-*analysis* feature, which will be useful if the user needs to analyze the same dataset repeatedly with varying parameters and algorithms. In this case, the user can choose the project folder and the settings are automatically listed in the input form. After necessary changes in the parameter setting, the user can re-analyze the dataset and the result will be stored as a sub-folder in the main project folder. In addition, VDAP-GUI offers a comprehensive results viewing window, in which the user can view all the results of the project without navigating away from VDAP-GUI. Lastly, this tool can be executed in multiple instances at the same time.

### Comparison with other tools

In the recent years, variant detection and annotation tools have been developed in the free as well as commercial domains (Table 1). SPANDx is one such pipeline which was developed using Linux bash script (Sarovich and Price 2014). This pipeline works similar to VDAP-GUI, and the authors have incorporated phylogenetic analysis too. However, this command line pipeline does not take care of the quality control/trimming of the raw sequences (Table 1). Another tool namely WEP, is a web-based software providing graphical interfaces for variant discovery from raw NGS data (D’Antonio et al. 2013). Practically, uploading of huge datasets generated by the high-throughput experiments like NGS is not convenient. In addition, the access of the WEP tool is restricted, the analysis is specific for or limited to human exome sequences generated only by the Illumina platform. More recently, fastq2vcf tool has been developed, which has features very close to VDAP-GUI (Gao et al. 2015). This PERL based pipeline supports multiple platforms and single/paired-end reads. However, fastq2vcf is a command line tool, and the user needs to edit the configuration file before starting any analysis. This apart, fastq2vcf does not provide the option for trimming of raw sequences. Similarly, the ngs-backbone tool, a command line driven tool (Blanca et al. 2011), is restricted to the analysis of transcriptome data. Differently, the SeqGene tool is designed to handle both transcriptome and whole-exome datasets (Deng 2011). However, this command line tool does not provide options for trimming of raw reads. GAMES stand-alone tool is capable of handling whole genome as well as whole-exome datasets and generates excellent reports (Sana et al. 2011). However, this tool has command line interface and does not give options to process raw data. Finally, we have compared VDAP-GUI features with CLCBio genomics workbench 7.0.3 (http://www.clcbio.com). This commercial software has excellent graphical interface compared to VDAP-GUI and is much more user-friendly and intuitive, compared to similar tools in free domain. The software uses a proprietary algorithm for variant detection. It is also capable of detecting copy number variation (CNVs). However, the highly expensive licensing is a major limiting factor for this tool. Finally, we have excluded few popular pipelines (e.g., GATK, Atlas2) from the comparison with VDAP-GUI, as they do not process .fastq files, but require aligned files in SAM/BAM format in the first step (DePristo et al. 2011; Evani et al. 2012).

**Table 1:** Feature comparison of variant detection and annotation pipelines

Features	VDAP-GUI	SPANDx	WEP	fastq2vcf	ngs_backbone	SeqGene	GAMES	CLCBio
Web-based/Stand-alone	Stand-alone	Stand-alone	Web-based	Stand-alone	Stand-alone	Stand-alone	Stand-alone	Web-based/stand-alone
Free for academic use	Yes	Yes	Yes (restricted access)	Yes	Yes	Yes	Yes	No
GUI	Yes	No	Yes	No	No	No	No	Yes
Organisms	No restriction	No restriction	Human	No restriction	No restriction	No restriction	No restriction	No restriction
Platforms supported	Illumina/454/ion torrent	Illumina/454/ion torrent	Illumina	Multiple	Multiple	Multiple	Multiple	Illumina/454/ion torrent
Mixed platform support	Yes	No	No	Yes	No	No	No	Yes
Paired-end support (PE)	Yes (PE for Illumina)	Yes (PE for Illumina)	Yes	Yes	Yes	Yes	Yes	Yes
Quality control	Yes	No	Yes	Yes	Yes	Yes	No	Yes
Sequence trimming	Yes (Default/Custom)	No	Yes	No	Yes	No	No	Yes (Default/Custom)
WGS	Yes	Yes	No	Yes	No	No	Yes	Yes
WES	Yes	Yes	Yes	Yes	No	Yes	Yes	Yes
RNA-seq	No	No	No	No	Yes	Yes	No	Yes
Tools for variant call	SAMtools/VarScan/Freebayes	SAMtools/GATK	GATK	SAMtools/GATK/SNVer	SAMtools/GATK	SeqGene SNP pileup	GAMES method	Proprietry method
Variant types	SNP/INDEL	SNP/INDEL	SNP/INDEL	SNP/INDEL	SNP/INDEL	SNP/INDEL/CNV	SNP/INDEL/CNV	SNP/INDEL/CNV
Annotation	Yes	Yes	Yes	Yes	Yes	Yes	Yes	Yes, limited
Reports	Yes	No	Yes	Yes (limited)	Yes (limited)	Yes (limited)	Yes	Yes

Integration of VEP annotation tool enables VDAP-GUI to annotate the variants from more than 65 eukaryotes.

The other widely used variant annotation tools are SnpEff and ANNOVAR (Cingolani et al. 2012; Wang et al. 2010). In VEP, protein functional consequence prediction is available for 10 organisms, including human, rat, cow, dog, etc.

VDAP-GUI is a unique combination of user-friendliness, customization and functionality. It can assist non-IT expert researchers who do not prefer to work with Linux command line. Notably, we have tested VDAP-GUI in Microsoft Windows 7 platform running with Virtual box software (https://www.virtualbox.org/).

### Test dataset and assessment of the pipeline

**Fig. 3:**
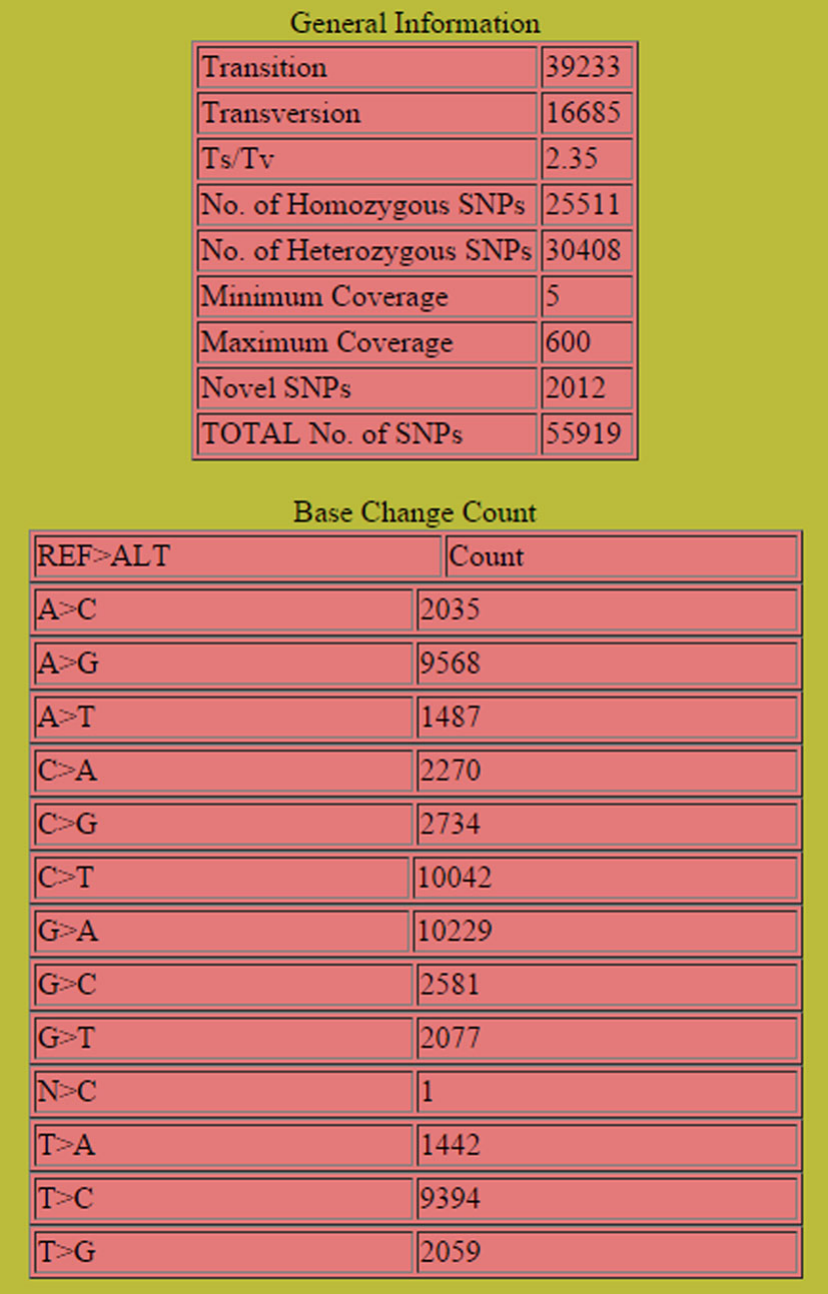
(*Summary of SNPs discovered in human whole*-*exome dataset*) the *upper panel* shows the general information of the Stage 1 detected SNPs such as Ts/Tv ratio, zygosity, number of SNVs, etc. The *lower panel* gives the frequency of SNPs in terms of nucleic acid changes. *REF* and *ALT* refers to bases in reference and sample, respectively, and *Count* indicates the frequency of each base change

We have considered a publicly available human whole-exome sequence dataset namely PDA_033-Tumor (NCBI SRA ID: SRX968817), which is a part of study titled “*Whole*-*exome sequencing of pancreatic cancer defines genetic diversity and therapeutic targets*” (Witkiewicz et al. 2015). The sequencing experiment had been performed using Illumina HiSeq2500 platform. The raw fastq files (paired-end) have been downloaded from NCBI SRA database to VDAP-GUI software along with the Human genome reference genome version hg19 (.fa file). In the workflow, we performed quality control of raw datasets (FastQC) and followed the default parameters for trimming (PRINSEQ) of Illumina paired-end NGS sequence dataset, i.e., phred quality score ≥20 and length ≥40 bp. The average coverage of the dataset was found to be about 36×. In VDAP-GUI, we chose variant type as ‘SNP’ and *analysis design*, with the default Stage-1 filter: depth = 5, quality = 20. SAMtools method was chosen for SNP discovery. With the given parameters, the pipeline discovered a total of 55,919 SNPs (homozygous SNPs = 25,511, heterozygous SNPs = 30,408) detected with transition-transversion (ts/tv) ratio of 2.35 (Fig. 3). Next, we loaded the resultant *.vcf* file to the *annotation* module of VDAP-GUI with Stage-2 filter criteria of depth = 10, quality = 30, to annotate the high-confidence SNPs, which resulted in the annotation of 46,963 SNPs (homozygous SNPs = 19,252, heterozygous SNPs = 27,712). Interestingly, out of 46,963 SNPs, 1537 have not been reported previously (Supplementary file 1 and 2). In an Ubuntu Linux-based desktop equipped with Intel i7 quad-core processor (8 MB L3 cache memory) and 16 GB RAM, the SNP discovery pipeline finished in 2 h and 44 min, and the annotation analysis took about 1 1/2 h till the generation of all annotation output files.

## Conclusions

VDAP-GUI is a simple and robust open-source tool developed primarily for non-IT expert researchers. It incorporates several proven tools at each step of variant discovery pipeline. Apart from the essential elements for variant discovery, VDAP-GUI’s unique features make it distinct in the non-commercial domain of variant discovery pipelines.

## Electronic supplementary material

Below is the link to the electronic supplementary material.

Supplementary material 1 (HTML 55 kb)

Supplementary material 2 (TXT 5211 kb)
