# Supplementary material for: VDAP-GUI: a user-friendly pipeline for variant discovery and annotation of raw next-generation sequencing data
Source: 3 Biotech. 2016 Feb 15;6(1):68. doi: 10.1007/s13205-016-0382-1 (PMC4754298; doi:10.1007/s13205-016-0382-1)
Supplement: Supplementary file 1 — Supplementary material 1 (HTML 55 kb) [file 13205_2016_382_MOESM1_ESM.html]

VEP summary


Links

- Top of page
- VEP run statistics
- General statistics
- Variant classes
- Consequences (most severe)
- Consequences (all)
- Coding consequences
- SIFT summary
- Variants by chromosome
- Position in protein

### VEP run statistics

|  |  |
| --- | --- |
| VEP version (API) | 78 (78) |
| Cache/Database | ./cache/vep//homo\_sapiens/78\_GRCh38 |
| Species | homo\_sapiens |
| Command line options | ``` --input_file ./WES_human_samtools_annotation_20150904_17:31/variant_filteration.vcf --cache --species homo_sapiens --sift b --check_existing --vcf --output_file ./WES_human_samtools_annotation_20150904_17:31/variant_annotation.vcf --dir ./cache/vep/ --offline --force_overwrite ``` |
| Start time | 2015-09-04 17:32:28 |
| End time | 2015-09-04 18:47:26 |
| Run time | 4498 seconds |
| Input file (format) | ./WES\_human\_samtools\_annotation\_20150904\_17:31/variant\_filteration.vcf (VCF) |
| Output file | ./WES\_human\_samtools\_annotation\_20150904\_17:31/variant\_annotation.vcf [text] |

### General statistics

|  |  |
| --- | --- |
| Lines of input read | 47025 |
| Variants processed | 46963 |
| Variants remaining after filtering | 46963 |
| Lines of output written | 46963 |
| Novel / existing variants | 1537 (3.3%) / 45426 (96.7%) |
| Overlapped genes | 21766 |
| Overlapped transcripts | 97860 |
| Overlapped regulatory features | - |

---

### Variant classes

---

### Consequences (most severe)

---

### Consequences (all)

---

### Coding consequences

---

### SIFT summary

---

### Variants by chromosome

---

### Distribution of variants on chromosome 1

---

### Distribution of variants on chromosome 2

---

### Distribution of variants on chromosome 3

---

### Distribution of variants on chromosome 4

---

### Distribution of variants on chromosome 5

---

### Distribution of variants on chromosome 6

---

### Distribution of variants on chromosome 7

---

### Distribution of variants on chromosome 8

---

### Distribution of variants on chromosome 9

---

### Distribution of variants on chromosome 10

---

### Distribution of variants on chromosome 11

---

### Distribution of variants on chromosome 12

---

### Distribution of variants on chromosome 13

---

### Distribution of variants on chromosome 14

---

### Distribution of variants on chromosome 15

---

### Distribution of variants on chromosome 16

---

### Distribution of variants on chromosome 17

---

### Distribution of variants on chromosome 18

---

### Distribution of variants on chromosome 19

---

### Distribution of variants on chromosome 20

---

### Distribution of variants on chromosome 21

---

### Distribution of variants on chromosome 22

---

### Distribution of variants on chromosome MT

---

### Distribution of variants on chromosome X

---

### Distribution of variants on chromosome Y

---

### Position in protein
